# Synthesis and Characterization of Poly(butylene glycol adipate-co-terephthalate/diphenylsilanediol adipate-co-terephthalate) Copolyester

**DOI:** 10.3390/polym16081122

**Published:** 2024-04-17

**Authors:** Tiejun Ge, Meiyuan Wang, Xiaofeng He, Yang Yu, Xiaofeng Liu, Bo Wen, Peihan Liu

**Affiliations:** 1 Department of Polymer Science and Engineering, Shenyang University of Chemical Technology, Shenyang 110142, China; getiejun@syuct.edu.cn (T.G.); 13166673858@163.com (M.W.); lxfchujian@163.com (X.L.); 2 Liaoning Polymer Materials Engineering and Technology Research Center, Shenyang 110142, China; 3 Liaoning Dongsheng Plastic Industry Co., Ltd., Yingkou 115003, China

**Keywords:** melt polycondensation, diphenylsilanediol, thermal stability, mechanical properties

## Abstract

The copolyester poly(butylene glycol adipate-co-terephthalate/diphenylsilanediol adipate-co-terephthalate) (PBDAT) was synthesized by the melt polycondensation method using terephthalic acid, adipic acid, 1,4-butanediol, and diphenylsilylene glycol as the raw materials. The molecular chain structure, thermal properties, thermal stability, mechanical properties, and degradation behaviors of the copolyesters were investigated. The results showed that the prepared PBDAT copolyesters exhibited good thermal stability and mechanical properties. With the increase in diphenylsilanediol (DPSD) content, the thermal stability of PBDAT and the melting temperature both increased. The tensile strength and elastic modulus of PBDAT also exhibited an increasing tend. When the DPSD content was 12.5% (PBDAT-12.5), the tensile strength, the elastic modulus, and elongation at break were 30.56 MPa, 238 MPa, and 219%, respectively. With the increase in diphenylsilanediol content, the hydrophilicity of PBDAT decreased, but PBDAT still shows good degradability and the thermal degradation T5% temperature was 355 °C. The thermal degradation of PBDAT was also improved.

## 1. Introduction

Since the rapid development of the plastics industry in the 1950s, plastics have been widely used in food packaging, agricultural mulch, construction, tissue engineering and other fields. According to statistics, the annual global production of plastics has reached 350 million tons and is increasing year by year [1,2,3,4]. Despite the convenience it brings to daily life and industrial production, the extensive use of plastics has also led to the consumption of resources and the accumulation of plastic waste, of which 12% is incinerated, 79% is landfilled or disposed of into the natural environment, and only 9% is recycled [2,5]. With the implementation of the plastic ban in recent years, biodegradable polymer materials that can be degraded to carbon dioxide and water in the natural environment have become a hot research topic [6]. Therefore, the development of biodegradable polymers, has gained increasing interest from both academia and industry [7]. Currently aliphatic–aromatic copolyesters are one of the most promising biodegradable polymers and have been developed for many years [8,9,10]. Poly(butylene adipate-co-terephthalate) (PBAT) is a popular and commercially viable biodegradable copolyester in terms of physical properties, processability and biodegradability as well as raw material price [11,12]. PBAT is a copolymerization of butylene terephthalate and butylene terephthalate. PBAT is considered one of the most promising polymers for biodegradable films for the food packaging industry [13]. PBAT is a synthetic aliphatic–aromatic copolyester, which fully degrades within a few weeks with the aid of enzymes naturally present in fertile soil. The aliphatic moiety is responsible for its biodegradability, and the aromatic part provides good mechanical properties compared to other polymers [14]. PBAT is a flexible plastic, designed for film extrusion and extrusion coating, has high elongation at break, as well as a good processability. Its mechanical properties are similar to those of polyethylene films [12,15]. It has been applied in the manufacture of agricultural films and laminated films for solid food packaging and garbage bags [16].

As an aliphatic–aromatic copolyester, PBAT possesses both the excellent mechanical properties of aromatic polyesters—good toughness, elongation at break, and impact resistance—as well as the biodegradability of aliphatic polyesters [17,18,19]. PBAT is suitable for injection molding, extrusion molding, blow molding and other processing methods, has a high elongation at break and often has linear low-density polyethylene (LDPE) substitutes, but its low tensile strength, low modulus of elasticity, as well as poor heat resistance limit the application of PBAT.

PBAT is usually modified by blending to enhance its properties [20,21,22] Samira et al. [23] found that the modified cellulose was uniformly dispersed in polyester after blending with PBAT. And the tensile properties and thermal stability of the composites can be significantly improved. Smita Mohanty et al. [24] used a variety of surfactants to modify montmorillonite clay and then seperated montmorillonite clay into PBAT to make nanocomposite membranes. The nanocomposite films prepared from bentonite (B190)-modified montmorillonite were tested to obtain the best mechanical properties. It was also found that the mechanical and thermal properties of the PBAT composite films grafted with maleic anhydride were higher than those of the unmodified ones. Barbosa et al. [25] found that the acetyl group improves the interaction between CNS and PBAT, which enhances the mechanical and thermal properties of the composites. The addition of cellulose reduces the cost and improves the mechanical properties, but it has an effect on the biodegradation rate of PBAT materials. S. Livi et al. [26] prepared composites using different ionic liquid-treated MMT filled with PBAT, which showed an increase in the tensile elastic modulus by up to 25%, but with a reduced barrier to water and CO_2_. Christina et al. [27] investigated the effect of the TPS content in the range of 50–70 wt% between TPS/PBAT blended films and their properties. The results showed that all the blended films had better thermal stability, tensile strength and modulus. However, the blending modification usually has the disadvantages of poor compatibility between materials and uneven dispersion between various substances. While copolymerization modification can effectively solve the problems brought by co-modification, there are fewer studies on copolymerization modification for PBAT [28,29,30]. Zhang Wei et al. [31] prepared PEF/PBAT block copolymers by esterification polycondensation, and the copolymers produced had good thermal stability, and the tensile strength, Young’s modulus and elongation at break were improved.

The main chain of silicone resin is composed of Si-O bond, and the bond energy of Si-O bond is larger than that of the C-C bond and the C-O bond, so the weathering and heat aging resistance of silicone resin are obviously better than that of epoxy resin [32]. Diphenylsilanediol is a very useful intermediate for the synthesis of silicone materials. Guo Hong et al. [33] used it to synthesize octaphenylcyclotetrasiloxane, which can be used to prepare silicone resins or inorganic–organic polymer silicone polymers directly by the condensation reaction [34,35]. Silicone-containing polymers have excellent mechanical and thermal properties as well as a variety of special optical properties [36]. Xu Jian et al. [37] chemically modified bisphenol A-type epoxy resin with diphenylsilylene glycol to obtain a new type of thermosolid epoxy resin with good heat, water and mechanical properties, which can be used for a long period of time under the condition of 250 °C; Kuang Wenfeng et al. [38] used diphenylsilylene glycol (DPSD)-modified bismaleimide (BMI) copolymerization resin to obtain a new type of engineering resin with good mechanical properties and thermal mechanical strength retention as well as high-temperature-resistant engineering plastic laminate. P. Madhusudhana Reddy et al. [39] modified organic–inorganic/graphene hybrid composite with DPSD as a sealant for white LEDs. The results showed that the refractive index of the film increased with the increase in DPSD content, and the thermal conductivity, thermal stability, and light transmittance were also improved; Shufang Jiang et al. [40] synthesized a series of diphenylsilanediol-modified epoxy resins and a new type of curing agent, and the results showed that the application of the modified resins and the newly synthesized curing agent resulted in the lowering of the rate of thermal decomposition of the cured products, the glass transition temperature (Tg) is only slightly reduced, the tensile modulus and tensile strength are improved, and the curing agent has higher thermal decomposition resistance due to the introduction of high-energy Si-O bonds. Therefore, the addition of DPSD to polyester can improve the heat resistance, thermal stability and thermal decomposition of the material

Therefore, in this study, a new copolyester PBDAT was synthesized by copolymerization modification of PBAT with DPSD, in which DPSD was introduced into the molecular chain of PBAT by the esterification polycondensation method, so as to enhance the mechanical and thermal properties of PBAT and broaden the use of PBAT. In this paper, a series of PBDAT with different DPSD contents were synthesized, and the chemical structures of the molecular chain of the copolyesters were characterized and the effects of different DPSD contents on the thermal properties, fluidity, molecular weight, thermal stability, mechanical properties and degradation properties of the copolymers were analyzed, which provided a theoretical basis for the chemical modification of PBAT and its applications.

## 2. Materials and Materials

### 2.1. Materials

Terephthalic acid (PTA): 99%; adipic acid (AA): 99%; 1,4-butanediol (BDO): 99%; diphenylsilanediol (DPSD):99%; tetrabutyl titanate (TBT): analytical pure; antimony trioxide (AT): analytical pure; stannous octanoate (Sn(Oct)): analytical pure; antioxidant 1010: analytical pure; 1,1,2,2-tetrachloroethane: analytical pure; phenol: analytical pure. All the above reagents are from Shanghai McLean Biochemical Technology Co., Ltd. (Shanghai, China) and are used as received.

### 2.2. Synthesis of PBDAT Copolyester

**Figure 1:**
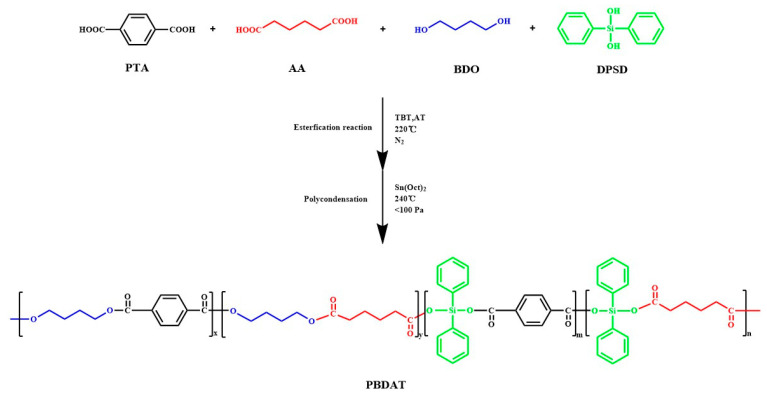
Synthesis of PBDAT.

In the first step, using the co-esterification method, BDO, AA, PTA, DPSD, and the esterification catalyst were added to the same 250 mL three-necked flask. The reaction temperature was 220 °C and when the water yield reaches 90% of the theoretical value, the esterification end point was taken. In the second step, the polycondensation catalyst stannous octanoate was added, and the temperature was raised to 240 °C. Then, the copolymerization was slowly evacuated to high vacuum (<100 Pa), when the pole-climbing phenomenon occurred until the stirrer power reached a maximum, the stirring was stopped, and then the material was discharged. The molar ratios of PTA and AA were fixed (total 0.3 mol, PTA:AA = 1:1), the molar ratio of alcohol to acid was controlled to be 1.4:1, the esterification temperature was set to 220 °C, the condensation temperature was set to 240 °C, the esterification catalysts (TBT, AT) were added at a quantity of 0.75% of the total mass of the dibasic acid, and the polycondensation catalysts (Sn(Oct)_2_) were added at a quantity of 0.75% of the total mass of dibasic acid, and the antioxidant 1010 was added at a quantity of 0.1 g. The synthetic route is shown in Figure 1. The copolyesters(1^#^–5^#^) corresponded to PBDAT-X (X = 2.5, 5, 7.5, 10, 12.5), and X is the percentage of DPSD to the molar ratio of the total alcohol amount.

### 2.3. Characterization

Fourier-transform infrared (FT-IR): The samples were made into rectangular slices, the surface was wiped with anhydrous ethanol, allowing the ethanol to evaporate, and the samples were tested by a Fourier-transform infrared spectrometer (Nicolet IS/10, Thermo Fisher Technologies, Waltham, MA, USA).

Nuclear magnetic resonance hydrogen spectroscopy (^1^H NMR): 15 mg of sample was taken with deuterated chloroform as the solvent, tetramethylsilane (TMS) as the internal standard, and the measurement temperature was 25 °C. The test was carried out by a solid–liquid nuclear magnetic resonance (NMR) instrument (500 MHZv, Bruker Instruments, Bremen, Germany).

Characteristic viscosity, viscosity-average molecular weight: The samples were dissolved in a solvent mixture of phenol: tetrachloroethane (3:2) at a concentration of 5 g/L. Tests were carried out using a viscometer with a capillary inner diameter of 0.88 mm and a thermostatic water bath at a temperature of 25 ± 0.05 °C. The viscosity was calculated using the following equation:

$$\eta_{r}=t_{1}/t_{0}$$

$$\eta_{sp}=\eta_{r}-1$$

$$\left[\eta \right]=\frac{\sqrt{1+1.4\eta_{sp}}-1}{0.7c}$$

where $\eta_{r}$ is the relative viscosity, $\eta_{sp}$ is the specific viscosity, *c* is the concentration of the sample, and *t*_0_ and *t*_1_ are the elution time of the solvent and solution, respectively.

The relationship between intrinsic viscosity and viscosity-average molecular weight is as follows:

$$\left[\eta \right]=KM\eta^{\alpha }$$

where *K* is the proportionality constant and *α* is the empirical parameter. *K* and *α* depend on temperature, polymer-solvent system, and molecular weight. In this paper, the *K* value is 2.1 × 10^−4^ dL/g, and the *α* value is 0.82.

The melt index (MI): The melt flow rate was tested according to GB/T3682-2000 [41] at 190 °C with a nominal load of 2.16 kg by the melt flow rate tester (ZRZ1452, Metus Industrial Systems Ltd., Sunnyvale, CA, USA).

Differential scanning calorimetery (DSC): A differential scanning calorimeter (Q-200, TA Company, Menlo Park, CA, USA) was used and tested according to GB/T 19466.3-2004 [42]. Under a nitrogen atmosphere, the heating rate was 10 °C/min, the upper limit of temperature was 230 °C, and the lower limit was −50 °C. The crystallinity is calculated according to the equation:

$$\mathrm{Xc}=\frac{\Delta \mathrm{Hm}\;}{\Delta \mathrm{Hf}\;}\times 100\%$$

where ΔHm is the melting enthalpy, ΔHf is the theoretical melting enthalpy of complete crystallization. According to the literature, the ΔHf of PBAT is 114 J/g [43], respectively.

Vicat softening point (VST): Using the Vicat softening point tester (RHWK-300, Jiangsu Weike Instrument Co., Ltd., Huai’an, China) and according to GB/T1633-2000 [44] to determine the Vicat softening point, using a force of 10 N, a heating rate of 120 °C/h, and a sample size of 10 mm × 10 mm × 4 mm, the temperature at which the needle is inserted at 1 mm is the Vicat softening point.

Thermal weight loss (TG): A thermogravimetric analyzer (DTG-60H, Shimadzu, Kyoto, Japan) was used to analyze the thermal stability of the product. In a nitrogen atmosphere, the temperature rises from room temperature to 800 °C at a rate of 10 °C/min. The test was completed when the sample cooled to below 40 °C.

Mechanical properties: According to GB/T1040-2006 [45], the mechanical properties were tested by universal electronic test (DF-101S, High-speed Rail Testing Instrument (Dongguan) Co., Ltd., Dongguan, China) with a tensile rate of 50 mm/min.

The water contact angle: The contact angle measuring instrument (JC2000E9, Shanghai Zhongchen Digital Technology Equipment Co., Ltd., Shanghai, China) was used to drop distilled water on the surface of the sample, and the five-point marking method was used to mark the highest point of the water drop and the left and right contact point between the wafer and the water drop.

Alkali degradation test: the specimen 5 g (±0.05) was added into the clean wide-mouth bottle with 50 mL of 2 mol/L NaOH solution, a group of samples were taken every two days, cleaned with deionized water, the surface was wiped, and placed in an electric vacuum drying oven at 40 °C for 48 h. The specimen was weighed after constant weight. The degradation rate of the specimen was calculated by the weight loss of the material:

$$\mathrm{Loss}\;\mathrm{of}\;\mathrm{weight}\;\%=\frac{W_{0}-W_{t}}{W_{0}}\times 100\%$$

where *W_t_* is the mass of the specimen after drying at degradation time *t* and *W*_0_ is the initial mass of the specimen.

## 3. Results and Discussion

### 3.1. The Chemical Structure of PBDAT Copolyester

**Figure 2:**
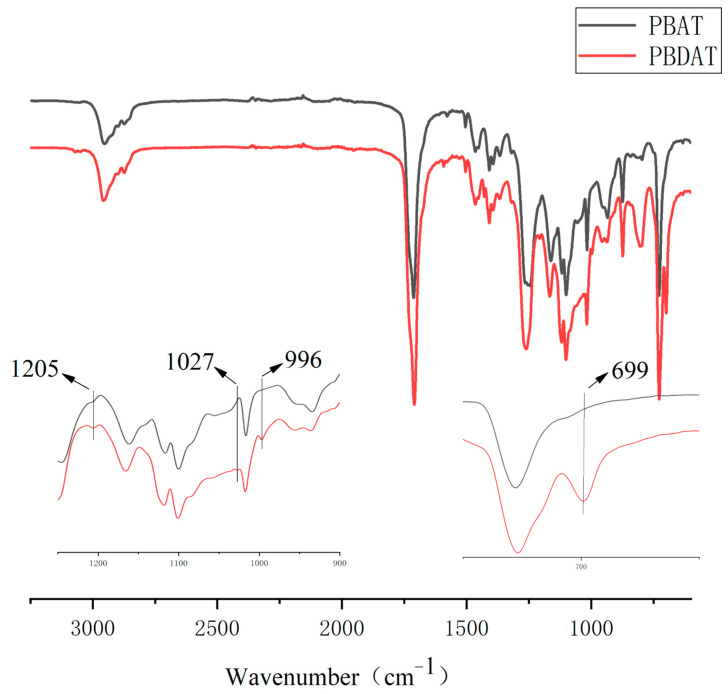
FTIR spectra of PBDAT.

Figure 2 shows the PBDAT infrared spectrum. As can be seen in Figure 2, the Si-C stretching vibration at 1205 cm^−1^, the C=O stretching vibration of the ester carbonyl group of PBCAT at 1710 cm^−1^, the stretching vibration of the benzene ring skeleton in the region of 1580~1410 cm^−1^, the stretching vibration of the ester C-O-C at 1250 cm^−1^, and the above absorption peaks indicate that the sample contains an ester group; the Si-O stretching vibration at 1027 cm^−1^; 996 cm^−1^ corresponds to the asymmetric Si-O-C stretching vibration; 728 cm^−1^ corresponds to the C-H bending vibration when the adipic acid chain segment is connected with four or more methylene.

**Figure 3:**
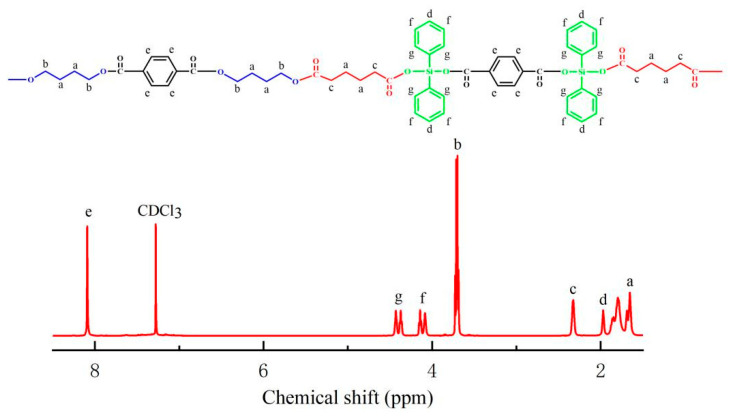
^1^H NMR spectra of PBDAT.

The structure of the polymer PBDAT was characterized by nuclear magnetic resonance hydrogen spectroscopy (^1^HNMR). Figure 3 shows the molecular formula and NMR hydrogen spectrum of PBDAT. As can be seen in Figure 3, the characteristic absorption peak of the solvent deuterated chloroform is at 7.26 ppm; the chemical shift is at 8.09 ppm (e), which corresponds to the resonance absorption of the four H atoms on the benzene ring of terephthalic acid; the chemical shift is at 1.68–1.65 ppm (a), which corresponds to the chemical shift with the signal that is indirectly connected to the carbonyl group in the adipic acid chain segment and to the ester group in the butanediol chain segment of the CH_2_ signals; (b) chemical shifts at 3.72–3.68 ppm correspond to CH_2_ signals directly linked to the ester group in the butylene terephthalate chain segment; (c) chemical shifts at 2.32 ppm correspond to signals linked to the carbonyl group in the adipic acid-containing chain segment; (d) chemical shifts at 1.97 ppm correspond to signals in the para-CH position on the benzene ring linked to Si; (f) chemical shifts at 4.15–4.08 ppm correspond to signals in the interstitial CH position on the benzene ring linked to Si; and (g) chemical shifts at 4.43–4.35 ppm are signals of neighboring CH on the benzene ring attached to Si. Combined with infrared spectra and NMR hydrogen spectra, it can be demonstrated that diphenylsilylene diols were joined the molecular chain segments, and PBDAT copolyesters were successfully prepared.

### 3.2. Fluidity and Processability of PBDAT Copolyesters

The characteristic viscosity [*η*] is used to characterize the internal friction effect between the polymer chains and the solvent molecules. As can be seen in Table 1, the characteristic viscosity of PBDAT copolyesters increases with the increase in the content of DT and DA units, with the highest [*η*] value of 2.54 dL/g for 5^#^. The viscosity-average molecular weight is in line with the trend of the characteristic viscosity, while the melt flow rate (MI) showed the changing trend in the opposite direction. The melt flow rate is an important index to describe the flow behavior and processing properties of polymers. In general, the higher the melt index, the better the flow of the polymer melt will be. As the DPSD content increases, the polymer molecular chain becomes rigid and the corresponding polyester flowability deteriorates, as evidenced by the change in the MI value from 2.83 g/10 min of 1^#^ to 2.54 g/10 min of 5^#^. When the DPSD content reaches 12.5%, the MI is 2.54 g/10 min, which is theoretically more suitable for extrusion processing.

### 3.3. Thermal Properties of PBDAT Copolyester

#### 3.3.1. DSC

**Figure 4:**
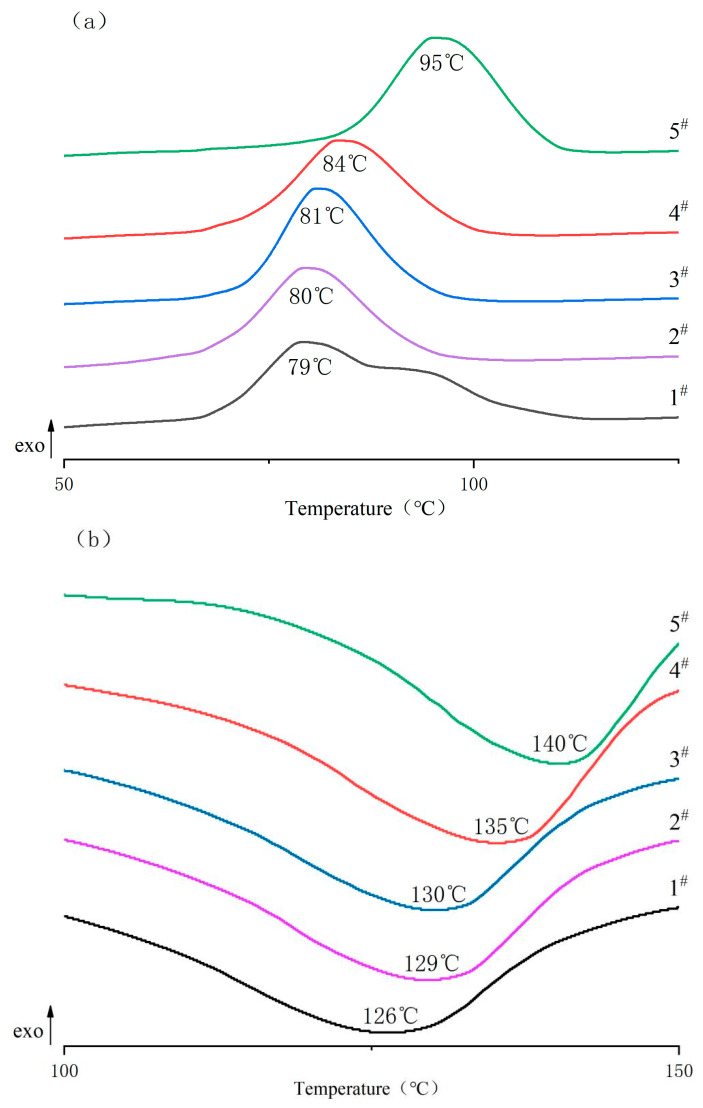
DSC curves of PBDAT copolyester: (**a**) cooling scanning and (**b**) secondary heating scanning.

The DSC curves of the PBDAT copolyesters are shown in Figure 4. The thermal transition data are summarized in Table 2.

The crystallization temperature (T_c_) is a reflection of the rearrangement of molecular chain segments in the amorphous region near the existing crystalline regions. For five samples containing diphenylsilanediol segments, T_c_ was proportional to the amount of DPSD added, and the T_c_ of PBDAT was higher than that of pure PBAT, indicating that the introduction of diphenylsilanediol chain has a significant effect on T_c_. The reason may be that the presence of diphenylsilanediol changed the regularity of the polyester molecular chain, and the single-chain rotation is difficult; at the same time, it can also be seen from the characteristic viscosity that the molecular weight of PBDAT is greater than that of the pure PBAT, and the molecular weight is an important factor of T_c_. On the other hand, the diphenylsilanediol existing in the molecular chain of PBDAT lead to positional resistances from the two benzene ring side groups, and symmetry of the distribution, which can reduce the internal rotational barriers.

The enthalpy change in melting (ΔH_m_) of PBDAT increased with the increase in DPSD content and was greater than that of pure PBAT. This is a reasonable reflection of the molecular structure of the polyester material, and it may be that the addition of silicon compounds affects the regularity of the polyester, leading to difficulties in the movement of the molecular chain, which makes the enthalpy of melting increase.

#### 3.3.2. VST

**Figure 5:**
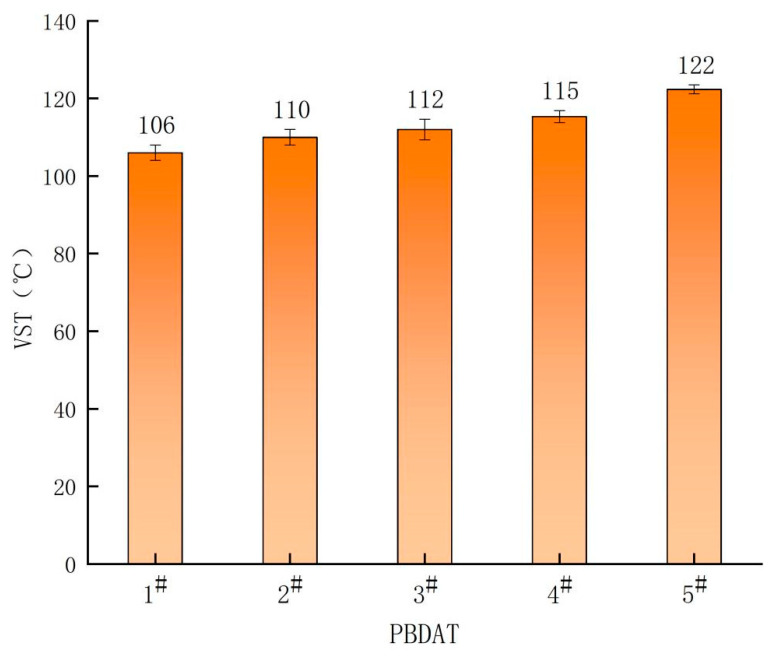
VST of PBDAT copolyesters.

Figure 5 shows the VST of PBDAT copolyesters with different DPSD content. It can be seen from the figure that the VST of the copolyesters increases from 106 °C of PBDAT-2.5 to 122 °C of 5^#^ with the increase in DT and DA components, which indicates that the introduction of the rigid benzene ring, a heat-resistant group, can greatly improve the heat resistance of the copolyesters, This is because the higher the proportion of DT and DA units, the greater the crystallinity, and the more energy is required to destroy this ordered structure, thus leading to effectively improve the heat resistance of the copolyester.

### 3.4. Thermogravimetric Analysis of PBDAT Copolyester

**Figure 6:**
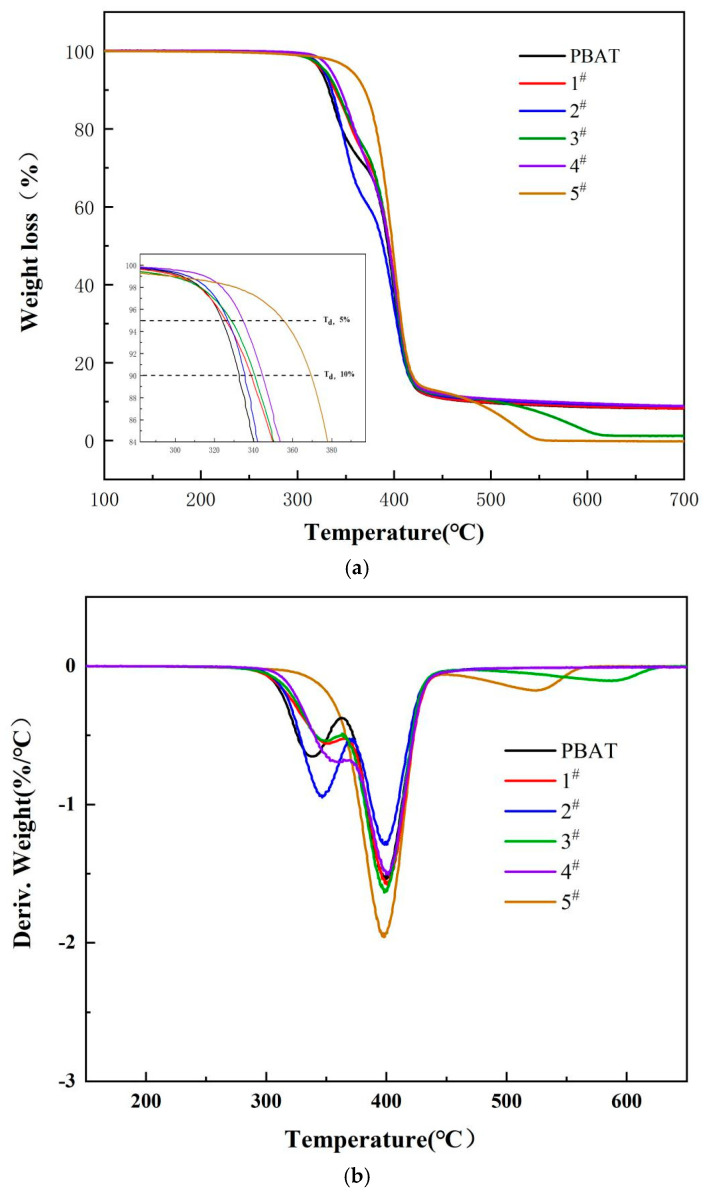
Thermogravimetric curves of PBDAT copolyester (**a**) TGA curves (**b**) DTG curves.

Figure 6 shows the TG curve (a) and the DTG curve (b) of PBAT and PBDAT. The thermal decomposition steps of PBAT and PBDAT with different DPSD contents were basically the same, and the heat weight loss process was the decomposition process of random copolymer, and the tested samples had good thermal stability. The initial decomposition temperature of PBAT was 324 °C. The initial decomposition temperatures of PBDAT were higher than that of PBAT, and the increase was approximately (1~9%), so that overall the thermal stability of PBDAT was better than that of PBAT. Therefore, in general, the thermal stability of PBDAT is better than that of PBAT. With the increase in DPSD content, the T_5%_ temperature of PBDAT increased from 327 °C to 355 °C. This means that the copolyester has a fairly high thermal stability. The maximum decomposition rate temperatures were all at 400 °C. The maximum decomposition rate temperatures were all at 400 °C. The main reason is that DPSD brings silicon-oxygen and silicon-carbon bonds with high bonding energy as well as high stability, and the increase in benzene ring content increases the proportion of aromatic chain segments and decreases the proportion of aliphatic chain segments, so it makes the T_5%_ temperature of PBDAT increase and better thermal stability.

### 3.5. Mechanical Properties of PBDAT Copolyester

**Figure 7:**
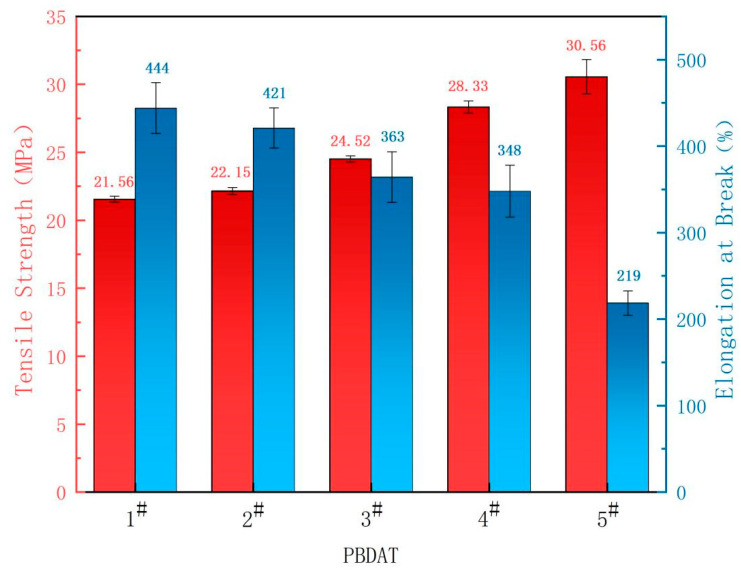
Tensile strength and elongation at break of PBDAT copolyester.

In general, molecular weight, chain flexibility and crystallinity all play an important role in the mechanical properties of copolyester. Chain flexibility and crystallinity are the two main factors to be considered. As shown in Figure 7 and Table 3, 5^#^ shows the highest modulus of elasticity (238 MPa) and tensile strength (30.56 MPa) due to the highest molecular chain stiffness and crystallinity. At the same time, the tensile strength and the elastic modulus of the polyester increased gradually with DPSD content increasing while the elongation at break decreased, because the rigid benzene rings in the side chain as well as the silicon-oxygen and silicon-carbon bonds brought by the DPSD make the molecular chain difficult to move and carry high bonding energies.

### 3.6. Degradation Properties of PBDAT Copolyester

**Figure 8:**
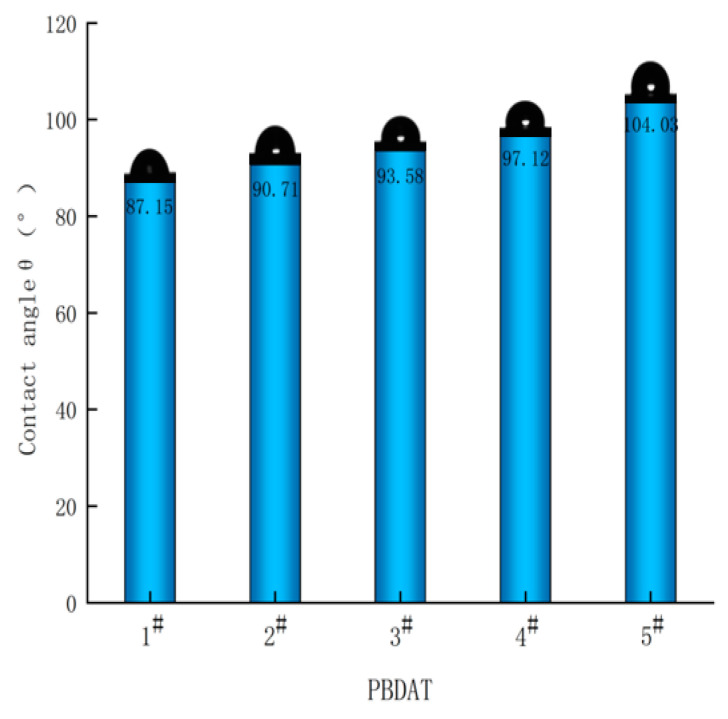
The water contact angle of PBDAT copolyester.

In nature, degradable materials usually need to be wet to undergo molecular degradation, so it is necessary to test the water contact angle to induce hydrophilicity in the material so as to indirectly reflect the possibility of the material to be eroded in a wet environment. Figure 8 shows the water contact angle graph of PBDAT. It can be seen that the water contact angle gradually increases from 87.15° to 104.03° with the increase in DPSD content, and the material becomes more and more hydrophobic, which will have a negative impact on the degradability of the material. The reason is due to the fact that the two side groups in the benzene ring introduced by DPSD, with non-polar bonds, greatly repel the contact of water molecules, thus making the water contact angle larger and the material more hydrophobic.

#### Alkali Degradation Experiment

**Figure 9:**
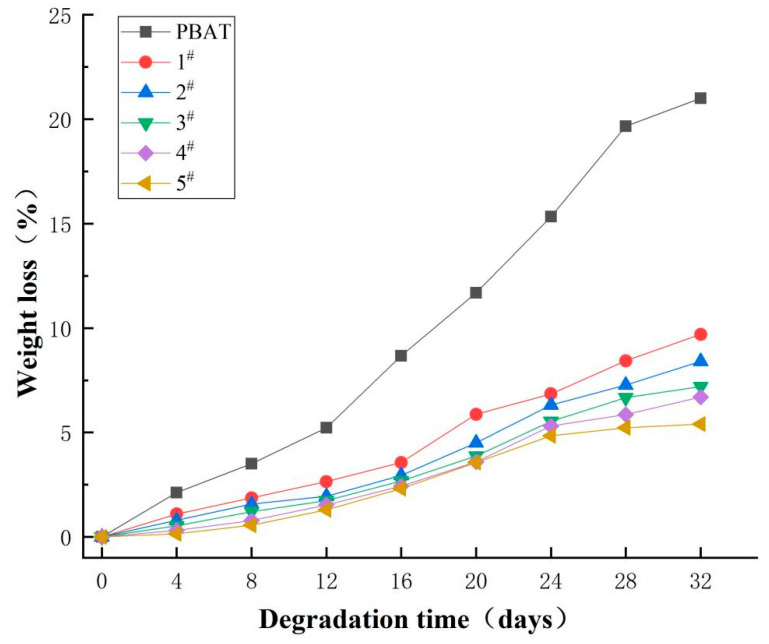
Alkali degradation of PBDAT copolyester.

Furthermore, as shown in Figure 9, the degradation of the material in lye was tested. Because the material is hydrophobic, it is difficult for water to infiltrate the surface of the material; therefore with the increase in the content of DPSD, the degradation rate of the material decreases—this conclusion is consistent with the result of water contact. Although the addition of DPSD affects the degradation rate of the material by the alkali, we can still ensure the degradation performance of the material. The maximum alkali degradation rate of PBDAT copolyester was 9.7% in 32 days.

## 4. Conclusions

PBDAT copolyester was successfully prepared by the esterification polycondensation reaction using AA, PTA, BDO, and DPSD as raw materials. PBDAT copolyester has good processing properties and excellent thermal stability. The tensile strength and the elastic modulus of PBDAT were both improved, reaching 30.56 MPa and 238 MPa, respectively, with an increase in DPSD content, which broadened the use of the copolyester. However, with the introduction of DPSD, the copolyester becomes less hydrophilic, but can still be degraded in alkaline solution. The highest alkali degradation rate reached 9.7% in 32 days, which is very friendly to the environment.The results are of guiding significance for the study of PBAT molecular chains and provide a new direction for the design, synthesis and development of new polyester materials.

## Figures and Tables

**Table 1:** Molecular weight and flow properties of copolyesters.

Samples	Characteristic Viscosity [η](dL/g)	Molecular Weight Average Mη (g/mol)	Melt Index MI (g/10 min)
1^#^	1.42	46,788	2.83
2^#^	1.46	48,320	2.77
3^#^	1.53	51,159	2.67
4^#^	1.58	53,675	2.60
5^#^	1.62	55,497	2.54

**Table 2:** Thermal properties of PBDAT copolyester.

Samples	T_m_/°C	T_c_/°C	T_g_/°C	ΔH_m_ (J/g)	ΔH_c_ (J/g)	X_c_ (%)
1^#^	126.26	79.16	−32.42	21.41	25.43	18.78
2^#^	129.43	79.38	−30.95	19.16	25.37	16.81
3^#^	129.95	80.81	−28.96	17.46	23.42	15.32
4^#^	135.28	84.61	−28.36	16.43	21.37	14.41
5^#^	140.32	95.25	−27.99	14.93	21.10	13.10

**Table 3:** Mechanical properties of PBDAT copolyester.

Samples	Tensile Strength (MPa)	Elongation at Break (%)	Modulus of Elasticity (MPa)
1^#^	21.56	444	159
2^#^	22.15	421	182
3^#^	24.52	363	196
4^#^	28.33	348	210
5^#^	30.56	219	238

## Data Availability

Data are contained within the article.
